# The Role of 6-Hour ECG in Patients with Left Bundle Branch Block After TAVI in Determining Same-Day Discharge

**DOI:** 10.3390/jcm14155408

**Published:** 2025-07-31

**Authors:** Muntaser Omari, Saif Memon, Debbie Stewart, Mohamed Ali, Richard Edwards, Rajiv Das, Timothy Cartlidge, Azfar Zaman, Mohamed Farag, Mohammad Alkhalil

**Affiliations:** 1 Cardiothoracic Centre, Freeman Hospital, Newcastle-upon-Tyne NE7 7DN, UK; 2 Translational and Clinical Research Institute, Newcastle University, Newcastle-upon-Tyne NE1 7RU, UK

**Keywords:** TAVI, LBBB, early discharge, self-expanding, balloon-expandable

## Abstract

**Background**: Left bundle branch block (LBBB) following trans-catheter aortic valve implantation (TAVI) has been excluded from same-day discharge. Early identification of patients with stable LBBB can help facilitate same-day discharge. We aim to assess the role of 6-hour ECG to determine development of LBBB in patients undergoing TAVI. **Methods**: This is a prospective single-centre study of patients who have LBBB following elective TAVI procedures. All patients underwent ECGs pre-TAVI, as well as immediately, 6 h, and 24 h post-TAVI. Changes in ECG were compared at 6 and 24 h with the one immediately post TAVI. **Results**: The study included 115 patients with uncomplicated procedures. The mean age was 81 ± 7 years, with 54% male. A self-expanding valve was used in 67% of patients. Following TAVI, prolongations of PR interval and QRS duration were dynamic and reduced at 6 h. The change in PR interval at 6 and 24 h was comparable [−11 (−20 to 3) vs. −2 (−24 to 16) ms, *p* = 0.18]. Similarly, there was no statistical difference in the change of QRS duration at 6 and 24 h compared to the ECG immediately post-TAVI [−10 (−40 to −2) vs. −7 (−34 to 0) ms, *p* = 0.055]. Changes in ECG were also comparable in patients undergoing balloon-expandable and self-expanding valves. **Conclusions**: The current study supports that 6-hour ECG has the potential to reduce the need for prolonged continuous monitoring post-TAVI. ECG at 6 h can help optimise patient flow and facilitate early discharge. Future studies with larger sample sizes are required to confirm our findings.

## 1. Introduction

Trans-catheter aortic valve implantation (TAVI) is increasingly considered the standard treatment for patients with severe symptomatic aortic stenosis (AS) with various surgical risks, including those who are at low risk [1,2,3]. Given its less invasive nature, TAVI has an advantage over surgical aortic valve replacement (SAVR) in reducing in-hospital length of stay and allowing early discharge of patients post procedure [4,5].

Reducing in-hospital length of stay by allowing patients to be discharged on the following day has become a popular strategy post TAVI [6,7]. Better operator experience combined with improvement in valve technology and delivery systems resulted in significant improvement in patient outcomes and procedural safety. This allowed the concept of daycase TAVI to emerge as a viable strategy to enable patients to be discharged from hospital on the same day following their procedure [8,9]. However, pre-existing conduction abnormalities remain a limiting factor in facilitating same-day discharge. More importantly, new changes in electrocardiogram (ECG) following TAVI can similarly preclude patients from being considered for same-day discharge. Overall, baseline or new conduction abnormalities on ECG are considered risk markers for developing high-degree atrioventricular blocks, which can lead to potential complications if patients are not optimally screened prior to hospital discharge [10].

Notably, left bundle branch block (LBBB) has not been consistently identified as a predictor of permanent pacemaker (PPM) [11]. Therefore, it is important to recognise that patients with LBBB may still be considered for early hospital discharge by ensuring that LBBB is not progressive. Features on electrocardiogram (ECG), such as prolongation of QRS complex or PR intervals following TAVI, can challenge such a strategy. A pre-emptive test that can reliably measure or predict progression of LBBB post-TAVI can help facilitate same-day discharge. The role of 6-hour ECG to determine development of LBBB has not been studied before and, if proven, has the potential to guide same-day discharge following TAVI with new or pre-existing LBBB.

## 2. Methods

**Figure 1:**
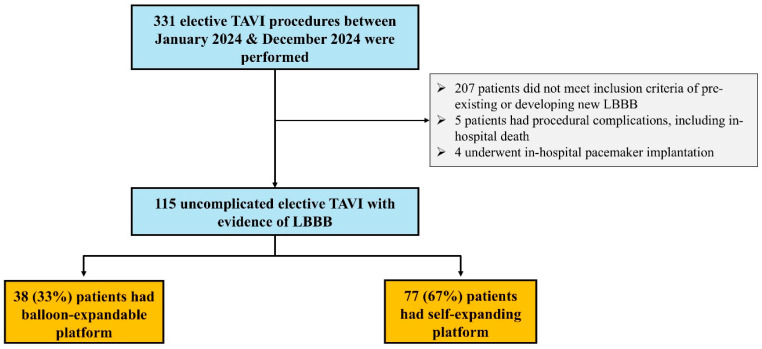
Study flow chart.

This was a prospective study of patients undergoing a TAVI procedure at a single UK centre between January 2024 and December 2024. Patients with severe native aortic stenosis or degenerative surgical bio-prosthesis and planned-for elective TAVI procedure were screened for inclusion in this study. Only patients with pre-existing or new-onset LBBB (QRS duration of more than 120 ms) were included in the current analysis. Patients who developed procedural complications, including the need for permanent pacemaker (PPM), within 6 h were excluded from the study. Additionally, those who had recent decompensated heart failure were also not included (Figure 1). A control cohort of patients without LBBB who underwent an elective TAVI procedure over the same period of time and met the inclusion and exclusion criteria were also included. A dedicated database was developed to enter patients’ clinical characteristics, and this database was monitored and maintained by a nurse specialist. Data were anonymised and formal ethics approval was not required for this specific study as part of a clinical audit, which was conducted in compliance with the Declaration of Helsinki.

Severe aortic stenosis was defined using transthoracic echocardiography, according to the current guidelines [1]. Patients with low flow state or impaired left ventricle function were included in this study, irrespective of the surgical risk. All patients underwent multi-detector computed tomography (MDCT) as part of their aortic stenosis work up to assess for suitability for transfemoral approach, as previously described [12,13]. Patients with prohibitive femoral access who underwent a non-transfemoral route, such as trans-axillary, were excluded from the study.

The TAVI procedure was performed by two experienced operators, according to the best practice. This included the use of ultrasound-guided puncture, minimal vascular access, and careful consideration for implant depth, as recommended [14,15]. The choice of the trans-catheter heart valve was left to the operator discretion.

Patients underwent ECG pre- and immediately post-TAVI. A third ECG was performed at 6 h and repeated at 24 h. Both ECGs at 6 and 24 h were compared to the ECG immediately post-TAVI to assess any difference in rate, rhythm, PR interval, and QRS duration. Both ΔPR interval and ΔQRS duration were compared between 6 or 24 h and immediately post-TAVI and labelled as follow:

ΔPR6H (difference in PR intervals between ECGs at 6 h and immediately post-TAVI)
ΔQRS6H (difference in QRS duration between ECGs at 6 h and immediately post-TAVI)
ΔPR24H (difference in PR intervals between ECGs at 24 h and immediately post-TAVI)
ΔQRS24H (difference in QRS duration between ECGs at 24 h and immediately post-TAVI)

Following TAVI procedure, patients were kept on continuous cardiac monitoring that was subsequently reviewed to determine hospital discharge. Patients who were discharged on the same day following TAVI were not excluded but were subsequently provided with a Holter monitor to assess for any evidence of high-degree atrio-ventricle block (HD-AVB). Additionally, they were contacted by a TAVI specialist nurse to assess symptoms and to ensure no clinical features suggestive of HD-AVB. This group had to meet specific criteria, which included uncomplicated trans-femoral TAVI procedure with satisfactory results, including less than moderate aortic regurgitation, fully ambulatory at 4 h post-procedure with no significant access-site bleeding or hematoma, and the presence of adequate social support for the patient during the first night post procedure.

Clinical outcomes, such as death from any cause, neurological events, or late PPM or vascular complications at 30 days, were recorded. Late PPM was defined as any PPM after TAVI for any indication that was not present at 6 h post-TAVI. Patients who had an indication for PPM within the first 6 h were excluded from the analysis, as highlighted above. Other endpoints were individual components of device safety at 30 days according to Valve Academic Research Consortium 3 (VARC-3) [16]. This includes vascular access-related complications, any bleeding requiring hospitalization, all stroke (or transient ischaemic attack).

### Statistical Analysis

Shapiro–Wilk test was used to assess data for normality. Normally distributed data were presented as mean and standard deviation and compared using unpaired *t*-test. On the other hand, data that were not normally distributed were presented as median and interquartile range and compared using Wilcoxon rank sum test. Categorical variables were presented as absolute number and percentages; and compared using χ^2^ test or Fisher’s exact test, as appropriate. Correlations between variables were assessed using Pearson or Spearman test, as appropriate. All statistical analyses were performed using SPSS 29.0 (SPSS, Inc. Chicago, IL, USA) and a *p* value of <0.05 was considered significant.

## 3. Results

Over the study period, 124 patients who underwent an elective TAVI procedure had pre-existing or developed new-onset LBBB. There were nine patients who were subsequently excluded as they developed in-hospital complications, including the need to insert PPM immediately post-TAVI (Figure 1).

The mean age was 81 ± 7 years, with 54% male. The mean gradient was 45 ± 12, and 92% had preserved left ventricle function on transthoracic echocardiography. Two patients had pre-existing pacemakers with underlying rhythm on ECG (results remained consistent after excluding those two patients). The median length of in-hospital stay was 1 (1–1.3) day, and 14 patients (12%) underwent same-day discharge following TAVI.

A self-expanding platform was used in 67% of patients. There were no differences in the baseline characteristics among patients who underwent self-expanding versus balloon-expandable valves, although diabetes was more frequently present in patients in the former group (33% vs. 13%, *p* = 0.027) (Table 1). Similarly, the severity of aortic valve disease, symptomatic status, and degree of left ventricle function was comparable between the two groups.

Immediately following TAVI, there was a significant increase in the duration of PR interval (180 ± 37 vs. 199 ± 42 ms, *p* < 0.001) and QRS duration (106 ± 26 vs. 146 ± 17 ms, *p* < 0.001) compared to baseline ECG. These changes were dynamic and the durations of PR interval and QRS duration decreased at 6 h post TAVI (*p* = 0.012 and *p* < 0.001, respectively) (Table 2). The change in PR intervals at 6 and 24 h was comparable [ΔPR6H −11 (−20 to 3) vs. ΔPR24H −2 (−24 to 16) ms, Wilcoxon Signed Ranks Test *p* = 0.18]. Similarly, there was no statistical difference in the change of QRS duration at 6 and 24 h compared to the ECG immediately post-TAVI [ΔQRS6H −10 (−40 to −2) vs. ΔQRS24H −7 (−34 to 0) ms, Wilcoxon Signed Ranks Test *p* = 0.055]. Finally, the difference in QRS duration between 6 and 24 h was small and measured as 2 (−4 to 7) ms, respectively.

**Figure 2:**
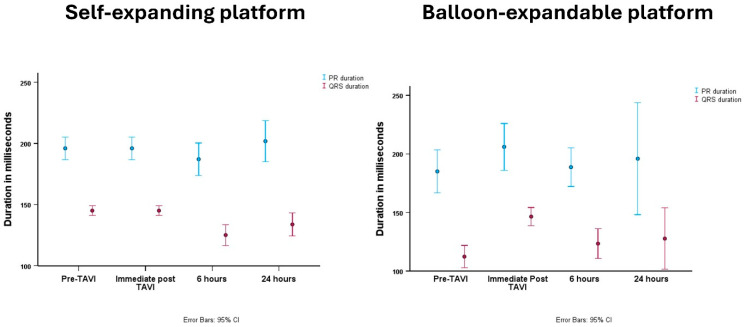
Changes in PR interval and QRS duration overtime, stratified according to the valve platform.

When comparing ECG changes according to the implanted platform, there were no differences in rate, rhythm, PR interval, or QRS duration at baseline ECG, immediately post-TAVI, at 6 h, or at 24 h between patients who underwent balloon-expandable versus self-expanding procedures (Table 2). The change in PR interval was comparable across valve platforms with no difference in patients receiving balloon-expandable (ΔPR6H −13 [−21 to 0] vs. ΔPR24H −14 [−24 to 3] ms, *p* = 0.44] and self-expanding valves (ΔPR6H −11 [−19 to 4] vs. ΔPR24H 3 [−27 to 20] ms, *p* = 0.13). There was no difference in the change in QRS duration in patients who received balloon-expandable valves (ΔQRS6H −10 [−36 to −3] vs. ΔQRS24H −10 [−40 to −4] ms, *p* = 0.77), with a better recovery at 24 h for those who received self-expanding valves (ΔQRS6H −10 [−40 to −2] vs. ΔQRS24H −6 [−26 to −1] ms, *p* = 0.012] (Figure 2).

**Figure 3:**
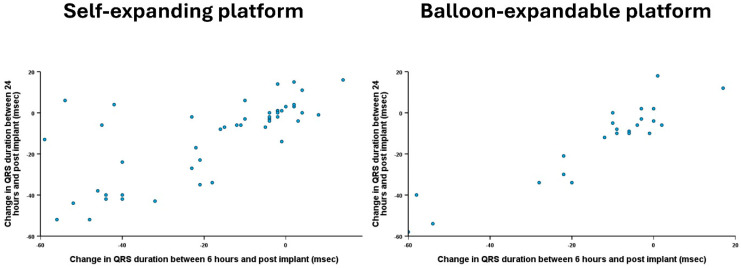
Scatter plot of individual patients highlighting change in QRS duration between 6 and 24 h compared to immediately post-TAVI and stratified according to the valve platform.

There was a strong association between ΔQRS6H and ΔQRS24H (Spearman r = 0.79, *p* < 0.001). This relationship was present in both groups who received self-expanding (Spearman r = 0.73, *p* < 0.001) as well as balloon-expandable valves (Spearman r = 0.89, *p* < 0.001) (Figure 3).

There was no death, but three patients underwent late PPM within one week following hospital discharge. All patients received self-expanding valves. On the binary logistic regression analysis, there was no echocardiographic or electrocardiographic parameter that predicted late pacemaker in this cohort. There were no cases of late vascular access-related complications or bleeding. There was no stroke reported up to one month following discharge. There was no difference in mortality rate at 30 days among elective patients with or without new or pre-existing LBBB (0% vs. 1%, *p* = 0.27) (both groups had comparable clinical and echocardiographic characteristics; and are presented in Table 3). Similarly, the composite of death, neurological events, late pacemaker, or vascular complications was comparable between the two groups [3 (2.4%) vs. 5 (2.9%), *p* = 0.80].

## 4. Discussion

The main findings from this study can be summarised as follow: (1) patients with pre-existing or new-onset LBBB can be considered for early hospital discharge; (2) 6 h ECG can be a used as a screening tool to assess progression of ECG changes post-TAVI; (3) such rules can be applied in patients undergoing balloon-expandable as well as self-expanding platforms.

Early hospital discharge in patients receiving TAVI is a significant advantage over patients undergoing SAVR. Over the last few years, physicians have developed protocols to allow safe and early hospital discharge, including same-day discharge [7,8,9]. The risk of conduction abnormalities following TAVI is a recognised complication and is frequently seen following procedure [17]. These changes have precluded patients from being considered for early discharge. Importantly, conduction abnormalities are commonly present prior to TAVI and their prevalence would inevitably contribute to the high frequency of conduction disturbances in TAVI studies [17]. It is recognised that LBBB is more frequently encountered in patients with severe compared to mild aortic stenosis [18]. In fact, there is four-fold increase in the prevalence of LBBB with more severe aortic stenosis and up to one in four patients undergoing TAVI had LBBB [11,18].

Previous studies reported new-onset LBBB can develop in up to 60% of patients following TAVI [19]. Certain procedural factors, such as implant depth and trans-catheter heart valve device, alongside other clinical or anatomical features, including membranous septum length, left ventricle outflow tract ellipticity, and calcium burden, are associated with developing LBBB [20,21]. Importantly, the development of LBBB is dynamic and potentially transient following TAVI. Previous data highlighted resolution of LBBB in up to 30% of patients prior to hospital discharge and further 40% by 30 days [17]. This phenomenon is applicable to both self- and balloon-expandable valves [22]. The prevailing hypothesis is attributed to the development and, subsequently, resolution of oedema surrounding the conduction system. The mechanical injury related to the deployment of the trans-catheter heart valve, including the use of balloon valvuloplasty, is perceived to be the main factor contributing to this phenomenon. Nonetheless, other mechanisms, such as the presence of calcium deposits, depth of implant, and baseline QRS changes, have also been highlighted [22,23]. Importantly, and unlike persistent LBBB, no predictors of transient LBBB were established [22].

There is conflicting evidence on the effect of new-onset LBBB and the development of HD-AVB [24,25]. A meta-analysis of first-generation TAVI devices highlighted a more than two-fold increase in the risk of PPM at one-year follow up [24]. More recently, Ream et al. reported the incidence of HD-AVB in 150 consecutive patients who underwent TAVI and received 30 days ambulatory event monitoring [25]. The authors demonstrated that new LBBB was not a risk marker for HD-AVB [25]. Although the sample size was relatively small, this study systematically assessed the early risk of HD-AVB in patients undergoing TAVI. Additionally, the median time for developing HD-AVB was 6 days following hospital discharge. Collectively, early discharge in patients with new-onset LBBB can be considered. Clinical assessment, procedural steps, and progression of ECG changes should all be factored in the decision making.

The presence of LBBB can be associated with reporting bias, leading to an increased rate of PPM. Fischer et al. [26] highlighted the increased risk of early PPM in patients with LBBB. This risk was not evident after 30 days. The Kaplan–Meier curves demonstrated parallel lines after the first few days. Therefore, pre-emptive PPM in patients with LBBB may be based on perceived, rather than actual, risk of HD-AVB [10,26]. In fact, this was the case in at least one of our patients in the study, whereby non-specific symptoms were attributed to HD-AVB and the patient underwent PPM. There is likely a Hawthorne effect at play in such cases, whereby demonstrating LBBB could bias interpretation of non-specific symptoms into inserting PPM.

Our study highlights the utility of 6-hour ECG in determining the duration of in-hospital stay for patients undergoing TAVI with pre-existing or new-onset LBBB. Our data do not propose 6 h ECG as a predictor of late PPM in patients with pre-existing or new-onset LBBB. We demonstrated that changes at 6 h are comparable to those at 24 h. Therefore, if clinical and procedural assessments would allow early hospital discharge, then the patient does not have to stay overnight for continuous monitoring. In other words, the 6 h ECG would negate the need to wait for the following day to decide about hospital discharge and can provide important information to guide decision making regarding same-day discharge.

We observed an increase in PR interval and QRS duration immediately following TAVI procedure. The change in PR interval was less than 20 ms, while the QRS duration was prolonged by almost 40 ms. Interestingly, these immediate changes were comparable among patients who received self-expanding and balloon-expandable valves. Importantly, our data exclusively included patients with pre-existing or new-onset LBBB and, therefore, such changes cannot be applied to all patients undergoing TAVI. Notably, prolonged PR interval and QRS duration were transient and improved at 6 h with no further changes at 24 h. This pattern was comparable across the used trans-catheter heart valve platform.

Our study has several limitations that need to be acknowledged. Firstly, this was a single-centre study that included elective patients with pre-existing or new-onset LBBB following TAVI procedures. Therefore, the results of the study cannot be extrapolated to patients who had recent decompensation and require urgent TAVI. Secondly, the number of patients within the study was relatively small, and although the study exclusively assessed patients with LBBB, it remains underpowered to adequately ascertain safety outcomes. The risk of type II error cannot be fully excluded, and larger sample size studies are needed in the future. Finally, the decision to insert PPM was not adjudicated and can be subjected to Hawthorne bias.

In conclusion, our study supports that 6 h ECG has the potential to reduce the need for prolonged continuous monitoring of heart rhythm post-TAVI. This simple readily available test can help in optimising patient flow and be utilised irrespective of the used trans-catheter heart valve platform. Future studies with larger sample sizes are required to confirm our findings.

## Figures and Tables

**Table 1:** Baseline clinical and echocardiographic characteristics of included patients.

	Whole Cohort (*n* = 115)	Self-Expanding Platform (*n* = 77)	Balloon-Expandable Platform (*n* = 38)	*p* Value
Age, yrs (mean ± SD)	81 ± 7	81 ± 6	80 ± 8	0.21
Male gender (*n*, %)	62 (54%)	38 (49%)	24 (63%)	0.16
Body mass index, kg/m^2^ (mean ± SD)	30 ± 6	30 ± 6	30 ± 7	0.95
Diabetes (*n*, %)	30 (26%)	25 (33%)	5 (13%)	0.027
Previous smoker (*n*, %)	41 (36%)	27 (35%)	14 (37%)	0.85
Obstructive lung disease (*n*, %)	4 (4%)	4 (5%)	0 (0%)	0.15
Creatinine, mmol/L (mean ± SD)	97 ± 37	92 ± 32	105 ± 43	0.11
Previous CVA/TIA (*n*, %)	4 (4%)	3 (4%)	1 (3%)	0.73
Atrial fibrillation (*n*, %)	26 (23%)	17 (22%)	9 (24%)	0.85
NYHA III/IV (*n*, %)	72 (63%)	46 (60%)	26 (68%)	0.37
Mean gradient, mmHg (mean ± SD)	45 ± 12	45 ± 13	44 ± 11	0.77
Peak gradient, mmHg (mean ± SD)	76 ± 19	76 ± 20	71 ± 17	0.97
Aortic valve area, cm^2^ (mean ± SD)	0.75 ± 0.47	0.80 ± 0.55	0.66 ± 0.21	0.27
Moderate MR (*n*, %)	2 (2%)	2 (3%)	0 (0%)	0.32
Preserved LV function (*n*, %)	106 (92%)	70 (91%)	36 (95%)	0.47
Same-day discharge (*n*, %)	14 (12%)	11 (14%)	3 (8%)	0.32

CVA: cerebrovascular event; LV: left ventricle; NYHA: New York Heart Association; MR: mitral regurgitation; SD: standard deviation; TIA: transient ischemia attack. Baseline transthoracic echocardiography parameters are reported within 4 months of the TAVI procedure.

**Table 2:** Procedural, electrical, and echocardiographic outcomes of included patients.

	Whole Cohort (*n* = 115)	Self-Expanding Platform (*n* = 77)	Balloon-Expandable Platform (*n* = 38)	*p* Value
Pre TAVI				
Sinus rhythm (*n*, %)	88 (77%)	59 (77%)	29 (76%)	0.77
Heart rate (mean ± SD)	73 ± 13	73 ± 14	72 ± 12	0.65
PR interval (mean ± SD)	180 ± 37	177 ± 32	185 ± 46	0.37
QRS duration (mean ± SD)	106 ± 26	102 ± 27	113 ± 24	0.051
Immediately Post TAVI				
Sinus rhythm (*n*, %)	88 (77%)	58 (75%)	30 (79%)	0.67
Heart rate (mean ± SD)	68 ± 13	69 ± 13	68 ± 13	0.94
PR interval (mean ± SD)	199 ± 42	196 ± 36	206 ± 53	0.30
QRS duration (mean ± SD)	146 ± 17	145 ± 16	147 ± 19	0.74
At 6 h post TAVI				
Sinus rhythm (*n*, %)	88 (77%)	59 (77%)	29 (76%)	0.77
Heart rate (mean ± SD)	70 ± 14	71 ± 14	70 ± 14	0.77
PR interval (mean ± SD)	188 ± 42	187 ± 45	189 ± 37	0.89
QRS duration (mean ± SD)	126 ± 28	127 ± 28	124 ± 29	0.69
At 24 h post TAVI				
Sinus rhythm (*n*, %)	91 (79%)	61 (79%)	30 (79%)	0.77
Heart rate (mean ± SD)	73 ± 13	73 ± 13	73 ± 13	0.87
PR interval (mean ± SD)	200 ± 51	201 ± 40	196 ± 71	0.75
QRS duration (mean ± SD)	130 ± 26	131 ± 24	129 ± 31	0.77
Change in PR interval at 6 h * (median, IQR)	−11 (−20, 3)	−11 (−19, 4)	−13 (−21, 0)	0.62
Change in QRS duration at 6 h * (median, IQR)	−10 (−40, −2)	−10 (−40, −2)	−10 (−36, −3)	0.48
Change in PR interval at 24 h ^⁋^ (median, IQR)	−2 (−24, 16)	3 (−27, 20)	−14 (−24, 3)	0.29
Change in QRS duration at 24 h ^⁋^ (median, IQR)	−7 (−34, 0)	−6 (−26, 1)	−10 (−40, −4)	0.17

* Difference between 6 h and immediately post TAVI. ^⁋^ Difference between 12 h and immediately post-TAVI.

**Table 3:** Clinical and echocardiographic characteristics of patients with and without LBBB.

	LBBB Cohort (*n* = 115)	No LBBB Cohort (*n* = 174)	*p* Value
Age, yrs (mean ± SD)	81 ± 7	81 ± 6	0.57
Male gender (*n*, %)	62 (54%)	110 (63%)	0.12
Body mass index, kg/m^2^ (mean ± SD)	30 ± 6	28 ± 6	0.009
Diabetes (*n*, %)	30 (26%)	33 (19%)	0.15
Previous smoker (*n*, %)	41 (36%)	70 (40%)	0.43
Obstructive lung disease (*n*, %)	4 (4%)	7 (4%)	0.81
Creatinine, mmol/L (mean ± SD)	97 ± 37	95 ± 41	0.76
Previous CVA/TIA (*n*, %)	4 (4%)	11 (6%)	0.29
Atrial fibrillation (*n*, %)	26 (23%)	39 (22%)	0.97
NYHA III/IV (*n*, %)	72 (63%)	105 (60%)	0.70
Mean gradient, mmHg (mean ± SD)	45 ± 12	46 ± 12	0.45
Peak gradient, mmHg (mean ± SD)	76 ± 19	73 ± 22	0.21
Aortic valve area, cm^2^ (mean ± SD)	0.75 ± 0.47	0.75 ± 0.17	0.91
Moderate MR (*n*, %)	2 (2%)	2 (1%)	0.67
Preserved LV function (*n*, %)	106 (92%)	160 (92%)	0.95

CVA: cerebrovascular event; LV: left ventricle; NYHA: New York Heart Association; MR: mitral regurgitation; LBBB: left bundle branch block; SD: standard deviation; TIA: transient ischemia attack. Baseline transthoracic echocardiography parameters are reported within 4 months of the TAVI procedure.

## Data Availability

Data are available from corresponding author on a reasonable request.
